# Dietary Adequacies among South African Adults in Rural KwaZulu-Natal

**DOI:** 10.1371/journal.pone.0067184

**Published:** 2013-06-25

**Authors:** Fariba Kolahdooz, Kerry Spearing, Sangita Sharma

**Affiliations:** Department of Medicine, University of Alberta, Aboriginal and Global Health Research Group, Edmonton, Alberta, Canada; Chancellor College, University of Malawi, Malawi

## Abstract

**Background:**

Food quality, determined by micronutrient content, is a stronger determinant of nutritional status than food quantity. Health concerns resulting from the co-existence of over-nutrition and under-nutrition in low income populations in South Africa have been fully recognized in the last two decades. This study aimed to further investigate dietary adequacy amongst adults in rural KwaZulu-Natal, by determining daily energy and nutrient intakes, and identifying the degree of satisfaction of dietary requirements.

**Methods:**

Cross-sectional study assessing dietary adequacy from 24-hour dietary recalls of randomly selected 136 adults in Empangeni, KwaZulu-Natal, South Africa.

**Results:**

Results are presented for men (n = 52) and women (n = 84) 19–50 and >50 years old. Mean energy intake was greatest in women >50 years (2852 kcal/day) and exceeded Dietary Reference Intake’s for both men and women, regardless of age. Mean daily energy intake from carbohydrates was 69% for men and 67% for women, above the Dietary Reference Intake range of 45–65%. Sodium was also consumed in excess, and the Dietary Reference Intakes of vitamins A, B12, C, D, and E, calcium, zinc and pantothenic acid were not met by the majority of the population.

**Conclusion:**

Despite mandatory fortification of staple South African foods, micronutrient inadequacies are evident among adults in rural South African communities. Given the excess caloric intake and the rising prevalence of obesity and other non-communicable diseases in South Africa, a focus on diet quality may be a more effective approach to influence micronutrient status than a focus on diet quantity.

## Introduction

South Africa is a multicultural country whose population consists of Caucasian, Asian, and black African peoples, with black African making up approximately 80% of the population [1]. In the rapidly transitioning black African population of South Africa, large shifts have occurred in dietary and lifestyle patterns [2]–[5]. A shift in cooking methods from boiling to frying, and replacement of some traditional foods with pre-packaged processed foods is resulting in greater consumption of energy dense foods often lacking in micronutrients [6]–[8]. Causal factors to this shift are due in part to the high cost and low availability of healthier food options [4].

In what is unique to many middle income countries including South Africa, under-nutrition and over-nutrition often coexist [7]. Therefore, metabolic disease risk is an emerging problem despite the prevalence of under-nutrition and infectious diseases [9]. The high intake of energy dense foods among low income populations in South Africa is a source of the accelerating emergence of public health problems such as obesity [10], and the rising burden of many other non-communicable diseases [5]. Previous studies have shown a link between intake of energy-dense foods and low micronutrient intakes in rural areas of South Africa. This pattern is mediated by the low cost of energy-dense foods [4], and reinforced by the high palatability of sugar and fat [11]. In South Africa in 2008 the prevalence of overweight and obesity was 65.4% and 31.3% respectively in both males and females [12].

Starting in the 1930’s, nutrition intervention programs in developing countries focused on protein-deficiency, due to a misunderstanding that lack of dietary protein was the biggest factor of malnutrition. By the mid-1970 s the focus had shifted to energy deficiency due to lack of sufficient food as the main cause of malnutrition. Results of the Nutrition Collaborative Research Support Program (NCRSP) conducted in the 1980 s, showed that micronutrient malnutrition is the main nutritional problem in the world [13] and it is believed that food quality, determined by micronutrient content, is a stronger determinant of nutritional status than food quantity [14]. Inadequate intakes of micronutrients in developing countries have been fully recognized in the last two decades. Micronutrient deficiencies in calcium, vitamin D, iron, selenium, folate, and vitamins C, A, E and B6 are seen in a large percentage of pre-menopausal black South African women [15]. A recent large South African study assessing micronutrient intakes among adults in rural and urban areas determined that in particular, calcium, iron, folate and vitamin B6 were low in the diets. The study also found that inadequate intake of vitamin A was of particular concern amongst women of childbearing age, and those living in rural over urban areas were most affected by all micronutrient deficiencies [16].

The present study aimed to further investigate dietary adequacy amongst the peoples of rural KwaZulu-Natal, by determining daily energy and nutrient intakes, and identifying the degree of satisfaction of dietary requirements. This data could be used for dietary intervention in this community or later be used to assess dietary changes over time.

## Materials and Methods

### Recruitment and Data Collection

The cross-sectional study was conducted in the rural communities of Empangeni, KwaZulu-Natal (KZN), South Africa. Empangeni is located 160 km north of Durban (the capital of KZN), and is within the local municipality of UMhlatuze, which has a rural population of 185,581 people [17].

Participants from villages surrounding Empangeni were randomly selected for the study by a community liason, with the use of housing maps. One adult member of each household was invited to participate and was notified of the study requirements. In total, 137 individuals who met our criteria outlined below agreed to participate. Everyone in the surrounding communities was of the same socioeconomic status living in rondavels. Only men and women who were the main food preparers and shoppers for their household were interviewed. People <19 years, and pregnant or breastfeeding women were excluded.

The methodology for the collection and analysis of dietary intake and composite dishes has previously been described in detail [18]. In brief, twenty-four hour recalls were collected from 137 community members and were conducted by locally trained field staff. Training took place at Izulu Orphan Projects (IOP) charity, and included practice interviews on IOP staff. Fieldworkers were trained and supervised during data collection by the PI (S.S.). Twenty-four hour recalls were collected during the face-to-face interviews from two separate groups of participants. One 24 hour recall was collected from the first group for the purpose of compiling a list of commonly consumed foods for the development of a quantitative food frequency questionnaire, and two 24 hour recalls were collected on non-consecutive days from the second group to determine dietary adequacy. Data was collected either in English or in the local language (Zulu). Interviewers asked systematic questions to assist participants in recalling all foods and drinks consumed in the previous 24 hour period. Three dimensional food models (Nasco, Fort Atkinson, WI, USA) and local household utensils (cups, bowls and spoons) were used to assist participants in reporting the amounts of foods consumed. Additionally, 16 composite dishes were collected in the study community. The collection and analysis of composite dishes has previously been described in detail [19].

### Ethics Statement

The study was approved by the Research Ethics Board of the University of Alberta. Participants taking part in the study were required to give written and signed consent.

### Statistical Analysis

All dietary data from the interviewer-administered 24-hour recalls were coded and analyzed using Nutribase version 9 (Cybersoft Inc., Pheonix), which calculated energy and nutrient intakes per person. The food composition tables in Nutribase [20] were updated to include the weighed local and commonly used recipes collected from the population. Participants in the first group, and any participants in the second group from which only one 24 hour recall was obtained, were included in the analysis because their exclusion did not considerably change the mean nutrient intake adjusted for within-person variance, which was calculated using the participants with multiple recalls. Daily energy and nutrient intakes were compared with the appropriate Dietary Reference Intakes (DRIs) or Adequate Intakes (AIs) for men and women ages ≥19 years. Dietary adequacy was calculated using the Estimated Average Requirements (EARs) and was determined using the specific EARs according to gender/age groups when available; AIs were used instead for dietary fiber, vitamin D and calcium [21]. The degree of satisfaction of dietary requirements was estimated by calculating the proportion of the population in the ranges of energy and nutrient adequacies (<70%, 70-<90%, 90-<110%, and >110%). Individuals falling in the 90% to 110% range of the Acceptable Macronutrient Distribution Ranges (AMDR) for energy and in the 90% to 110% range of the EAR for nutrients are considered to have adequate intake of that nutrient. Consumption of adequate intakes or above is, therefore, defined as consuming >90% of AMDR for energy or >90% of EAR for nutrients. Nutrient intake data were not normally distributed and therefore non-parametric tests were used to compare the mean of nutrient intakes according to sex/age groups. All analyses were undertaken using SAS version 9.3 (SAS Institute, Inc., Cary, NC).

## Results

Dietary data was collected for 137 rural men and women in KwaZulu-Natal, South Africa. One subject was excluded from the analysis because of extreme mean energy intakes of >5000 kcal. Table 1 describes the subject characteristics of 52 males (mean age 43.2±16.6 years), and 84 females (mean age 50.8±14.9 years). A total of 39% of the males smoked compared to only 3% of the females. About 86% of females reported consuming supplements and only 2% of males did. Eight percent (8%) of males reported having a chronic condition such as hypertension, diabetes, and/or HIV; however, 15% reported taking medications for a chronic condition. From the females, 29% reported having one or more of these chronic disease conditions, yet 43% reported taking medications.

**Table 1:** Characteristics of rural men and women in KwaZulu-Natal, South Africa (n = 136).

Characteristics	Males(n = 52)	Females(n = 84)	p-values
**Age [mean (SD)]**	43.2 (16.6)	50.8 (14.9)	0.008
	**n (%)**	**n (%)**	
**Smoke**			
Yes	20 (39)	2 (3)	
No	32 (61)	82 (97)	0.0001
**Chewing Tobacco**			
Yes	1 (2)	3 (4)	
No	51 (98)	81 (96)	0.6
**Change in Diet**			
Yes/More	4 (8)	4 (5)	
Yes/Less	6 (12)	11 (13)	
No	42 (80)	69 (82)	0.5
**Supplement**			
Yes	1 (2)	72 (86)	
No	50 (80)	12 (14)	0.3
**Disease conditions** a			
Hypertension	1 (2)	5 (6)	
Diabetes	2 (4)	2 (2)	
HIV	1 (2)	8 (10)	
Multiple conditions (>1)	0	9 (11)	
No disease	50 (96)	60 (71)	0.1
**Medications**			
Blood pressure	2 (4)	13 (15)	
Diabetes	1 (1)	2 (2)	
Hypertension/other	5 (10)	22 (26)	
No medications	44 (85)	47 (55)	0.01

SD, standard deviation,

a Self reported information.

Table 2 describes the mean (SD) daily energy and nutrient intake of 136 South African adults in KwaZulu-Natal. The mean intake for energy exceeded DRIs for both men and women (2200 kcal and 1800 kcal respectively), regardless of age, with women >50 years consuming the most energy with a mean (SD) of 2852 (670) kcal/day. The mean percent daily energy intake from carbohydrates was approximately 69% for men and 66% for women, above the DRI range of 45–65%. The mean percent energy intake from protein was at the lower end of the DRI range (10–35%), at approximately 13% for men and 11% for women, and the mean percent energy from fat was approximately 18% for both men and women, below the DRI range of 20–35%. The mean intake of dietary fiber was above DRI (25 g) for both men and women in both 19–50 years and >50 years age groups at 36 g and 28 g respectively for men, and 39 g and 47 g respectively for women. Based on the mean intake, participants met the DRI for thiamine, riboflavin, niacin, Vitamin B-6, total folate, iron, and selenium. Significant difference (p<0.05) of mean nutrient intake between men and women ages 19–50 years was found for % energy from protein and vitamin A. For men and women ages >50 years, significant difference (p<0.05) in nutrient intake was seen for vitamin C and vitamin E. The mean intake for vitamins A, B-12, C, D, and E, pantothenic acid, calcium, and potassium did not meet the DRI. Women in both age groups met the DRI for zinc (8 mg), based on mean intake, however mean intake for men in both age groups did not meet the DRI of 11 mg. The mean sodium intake exceeded the DRI’s by 600 mg–800 mg across all age and gender groups.

**Table 2:** Mean (SD) daily energy and nutrient intake among South African adults in rural KwaZulu-Natal.

	19–50 years [Mean (SD)]			>50 years [Mean (SD)]			Dietary Reference Intake	
Nutrients	Men (n = 33)	Women (n = 40)	p-values	Men (n = 18)	Women (n = 44)	p-values	Men	Women
**Energy (kcal)**	2657 (879)	2774 (756)	1.0	2589 (1218)	2852 (670)	0.06	*2200*	*1800*
**% of energy from protein**	13 (3)	11 (2)	0.004	13 (3)	12 (3)	0.2	*10–35*	*10–35*
**% of energy from CHO**	69 (13)	67(12)	0.8	68 (9)	64 (11)	0.09	*45–65*	*45–65*
**% of energy from fat**	19 (11)	17(9)	0.7	18 (10)	17 (7)	0.8	*20–35*	*20–35*
**Protein (g)**	75 (34)	70(27)	0.2	60 (63)	71 (28)	0.5	*56*	*46*
**Carbohydrate (g)**	474 (174)	463 (159)	0.9	422 (184)	479 (146)	0.2	*130*	*130*
**Sugars (g)**	35 (25)	47 (24)	0.06	39 (53)	47 (21)	0.1	*<25% of energy*	*<25% of energy*
**Dietary fiber (g)^a^**	36 (18)	39(14)	0.7	28 (25)	47 (14)	0.07	*38*	*25*
**Fat (g)**	32 (22)	32(20.7)	0.6	25 (37)	31 (26)	0.3	*–*	*–*
**Saturated fat (g)**	6.3 (6)	7.4 (4.1)	0.8	6.9 (16.8)	8.8 (5.7)	0.3	*<10% of energy*	*<10% of energy*
**Monounsaturated Fat (g)**	12.8 (11.6)	14.5 (8.5)	0.7	10.6 (12.1)	16.3 (11.7)	0.08	*–*	*–*
**Polyunsaturated Fat (g)**	10.1 (7.6)	11.7 (6.0)	0.5	8.2 (7.6)	11.4 (8.7)	0.2	*–*	*–*
**Omega-3 fatty acid (g)**	0.01 (0.71)	0.01 (0.98)	0.4	0 (0.14)	0.01 (1.06)	0.7	*1.6*	*1.1*
**Omega-6 fatty acid (g)^b^**	0.01 (0.71)	0 (0.15)	0.06	0 (0.36)	0.01 (0.18)	0.07	*17*	*12*
**Cholesterol (mg)**	95 (87)	53.0(109)	0.6	91 (196)	74 (103)	0.6	*ALAP ^c^*	*ALAP*
**Vitamin A (µg-RAE)^d^**	125 (246)	216 (336)	0.02	134 (426)	196 (204)	0.2	*900*	*700*
**Thiamin (mg)**	3.1 (1.3)	2.6 (1.2)	0.7	2.8 (1.5)	3.1 (1.1)	0.3	*1.2*	*1.1*
**Riboflavin (mg)**	2.0 (0.9)	1.9 (0.9)	0.8	2.1 (1.9)	2.3 (0.8)	0.3	*1.3*	*1.1*
**Niacin (mg)**	30(13)	28 (12)	0.5	29 (20)	31.8 (11.0)	0.2	*16*	*14*
**Pantothenic Acid (mg)**	4.2 (1.9)	3.7 (1.4)	0.3	3.8 (4.5)	3.8 (1.5)	0.9	*5*	*5*
**Vitamin B-6 (mg)^e^**	1.7 (0.9)	1.8 (0.6)	0.8	2.2 (1.2)	1.8 (0.6)	0.9	*1.3*	*1.3*
**Total folate (µg-DFE)**	1633 (732)	1429 (793)	0.9	1532 (923)	1763 (716)	0.3	*400*	*400*
**Vitamin B-12 (µg)**	1.3 (4.8)	1.1 (6.2)	0.6	1.1(4.1)	1.5 (6.7)	0.6	*2.4*	*2.4*
**Iron (mg)^f^**	27 (12)	24 (10)	0.5	23 (15)	29 (15)	0.1	*8*	*18*
**Vitamin C (mg)**	20 (93)	38.1 (40.5)	0.1	20(37)	44 (45)	0.03	*90*	*75*
**Vitamin D (IU)^g^**	7.5 (102)	9.0 (128)	0.3	7.7 (32.1)	8.8 (149)	0.7	*15*	*15*
**Vitamin E (mg)^h^**	8.2 (7.0)	10.4 (5.3)	0.1	8.1 (6.0)	10.9 (8.4)	0.04	*15*	*15*
**Calcium (mg)^i^**	329 (188)	245 (206)	0.06	299 (1009)	265 (208)	0.9	*1000*	*1000*
**Magnesium (mg)**	369 (179)	393 (133)	0.5	331 (287)	409 (154)	0.4	*420*	*320*
**Potassium (mg)**	2511 (1442)	2442 (841)	0.4	2174 (2306)	2663 (1021)	0.4	*4700*	*4700*
**Sodium (mg)^j^**	2057(1199)	2100 (730)	0.7	2112 (1738)	2292 (843)	0.4	*1500*	*1500*
**Selenium (µg)**	99 (50)	102(44)	0.5	89 (60)	95 (44)	0.4	*55*	*55*
**Zinc (mg)**	8.6 (5.6)	8.3 (3.6)	0.4	8.2 (7.6)	9.3 (3.9)	0.5	*11*	*8*

CHO, carbohydrates; DRI, dietary reference intake; SD, standard deviation; ^a^ DRI for men >50 years 25 g/day and for women >50 years old 21 g/day; ^b^AI for men >50 years 14 g/day and for women >50 years old 11 g/day; ^c^As low as possible; ^d^As retinol activity equivalents (RAEs); ^e^DRI for men >50 years 1.7 mg/d; ^f^ DRI for women >50 years old 8 mg/day; ^g^As cholecalciferol; ^h^As α-tocopherol; ^i^DRI for women >50 years old 1200 g/day; ^j^DRI for men and women >50 years old 1300 mg/day.

The distribution of the range of dietary adequacy in key micronutrients for both men and women is described in table 3. A total of 24% of men and 7% of women had adequate intakes of energy, however the majority of women (77%) consumed >110% AMDR. Men’s energy consumption was more diverse, as 34% consumed <70% AMDR and only 18% consumed >110% AMDR. Seventy-one percent (71%) of men and 91% of women either met or exceeded the adequate intake (>90% EAR) for protein. Iron and selenium were both consumed in adequate intakes or above by 96% and 78% of men, respectively, and 79% and 95% of women, respectively. The majority of participants (67%–97%) had adequate intakes or above for thiamin, riboflavin, niacin, total folate and vitamin B-6. Nine-two percent (92%) of males and 86% of females consumed <70% the EAR for vitamin A, and 45% of males and 43% of females consumed <70% EAR for pantothenic acid. Sixty-one percent (61%) of males and 58% of females consumed <70% EAR for vitamin B-12, while only 37% and 32% of males and females, respectively, met or exceeded adequate intakes for vitamin B-12. Vitamins C, E, D, and calcium were inadequately consumed by the majority of the participants: less than 70% EAR was met by 86% and 62% of males and females, respectively, for vitamin C, 59% and 50% of males and females, respectively, for vitamin E, 98% and 96% of males and females, respectively, for vitamin D, and 92% and 96% of males and females, respectively, for calcium. Seventy-two percent (72%) of females, but only 41% of males, consumed adequate or greater daily intakes of zinc.

**Table 3:** Distribution of the range of dietary adequacy of energy and key micronutrients among men and women in rural KwaZulu-Natal, South Africa.

	<70% DRI^a^		70–90% DRI^a^		90–110% DRI^a^		>110% DRI^a^		
Nutrients	Men n (%)	Women n (%)	Men n (%)	Women n (%)	Men n (%)	Women n (%)	Men n (%)	Womenn (%)	p-values
**Energy (kcal)**	10(19)	2(2)	3(6)	2(2)	8(15)	6(7)	31(60)	74(88)	0.0001
**Protein (g)**	7(17)	3(4)	8(15)	4(5)	4(8)	7(8)	33(63)	70(83)	0.002
**Vitamin A (µg-RAE)** b	45(87)	59(70)	3(6)	7(8)	(0)	8(10)	4(8)	10(12)	0.05
**Thiamin (mg)**	2(4)	1(1)	2(4)	0(0)	2(4)	2(2)	46(88)	81(96)	0.06
**Riboflavin (mg)**	3(6)	1(1)	5(10)	2(2)	4(8)	2(2)	40(77)	79(94)	0.005
**Niacin (mg)**	3(6)	2(2)	2(4)	0	2(4)	0(0)	45(86)	82(98)	0.04
**Pantothenic Acid (mg)**	23(44)	36(43)	6(12)	23(27)	8(15)	14(17)	15(29)	11(13)	0.1
**Vitamin B-6 (mg)**	5(10)	4(5)	9(17)	2(2)	3(6)	9(11)	35(67)	69(82)	0.01
**Total folate (µg-DFE)**	2(4)	1(1)	1(2)	1(1)	0(0)	1(1)	49(94)	81(96)	0.4
**Vitamin B-12 (µg)**	30(58)	4(51)	1(2)	11(13)	1(2)	3(4)	20(38)	27(32)	0.9
**Iron (mg)**	0(0)	1(1)	2(4)	0(0)	0(0)	1(1)	50(96)	82(97)	0.6
**Vitamin C (mg)**	39(75)	44(52)	5(10)	10(12)	2(4)	5(6)	6(12)	25(30)	0.006
**Vitamin D (µg)b**	51(98)	71(92)	0(0)	1(1)	0(0)	1(2)	1(2)	5(6)	0.2
**Vitamin E (mg)** c	27(52)	24(29)	2(4)	18(21)	8(15)	7(8)	15(29)	35(42)	0.07
**Calcium (mg)**	44(85)	76(90)	5(8)	2(2)	2(4)	3(4)	1(2)	3(4)	0.8
**Selenium (µg)**	1(2)	2(2)	7(13)	1(1)	3(6)	1(1)	41(79)	80(95)	0.02
**Zinc (mg)**	8(15)	6(7)	9(17)	6(7)	5(10)	16(19)	30(58)	56(67)	0.05

DRI, Dietary Reference Intake.

a Acceptable Macronutrient Distribution Ranges (AMDR) for energy and Estimated Average Requirement (EAR) for nutrients.

b As retinol activity equivalents;

c As α-tocopherol.

## Discussion

We found that mean intake for energy exceeded DRIs for both men and women, and this pattern was most evident for females, as the majority of them consumed more than 110% the DRI of 1800 kcal/day. In addition to excess calorie consumption, we also found that ∼67% of this energy was provided by carbohydrates. The health implications for high-calorie, high-carbohydrate diets have been well documented [22], [23]. Diets high in carbohydrates, particularly refined carbohydrates, have known associations with high triglycerides, LDL levels and reduced HDL and insulin resistance, all contributing factors to obesity and CVD incidence [22]–[24].

Although the majority of the study population consumed adequate intakes of protein, most protein was obtained from plant-based foods. Despite adequate intakes of protein, there is a correlation between diets lacking in animal source foods (ASF) and micronutrient deficiencies, including iron, zinc, vitamin B-12, riboflavin, calcium and vitamin A [25]. USDA indicates that animal products provide 77% of calcium, 56% of zinc, and19% of iron in the U.S. diets [26]. Our data show inadequate intakes of vitamins A and B-12, calcium and zinc (for men). The adequate intakes of iron and riboflavin observed may be a result of the mandatory fortification of staple foods in South Africa with these key micronutrients [16]. The Nutrition Collaborative Research Support Program (NCRSP) assessed the dietary adequacy of children in particularly malnourished regions of Kenya, Mexico and Egypt and results show that intakes of animal-source energy and protein were positively correlated with intakes of vitamins A and B-12, riboflavin, and calcium. Strong statistical associations were reported between the intake of ASF and better growth, cognitive function, activity, pregnancy outcome and morbidity. Intake of ASFs was therefore determined to be the strongest predictor of functional capacity in children [27].

Supplementation and fortification are the most common strategies used to target micronutrient deficiencies, due to their relative ease of delivery and cost-effectiveness [14]. Maize and wheat flour are the staple foods in South Africa, and in 2003 the South African government introduced mandatory fortification of these foods with vitamin A, iron, zinc, folic acid, thiamine, riboflavin, niacin, and vitamin B6. A study by Steyn et al. 2008 evaluated the effects of fortification on selected nutrient intakes. Fortification was found to raise mean levels of thiamine, riboflavin, niacin, vitamin B6 and folate above the recommended nutrient intakes. Although the fortifications positively increase the micronutrient profile of rural South Africans, some of the fortified micronutrients, including zinc and vitamin A, remain below adequate intake [16].

Despite fortification we found that 72% of females, but only 41% of males, consumed adequate daily intakes of zinc. These results could be due in part to the low intake of ASF. Zinc in ASF is more bioavailable, as plant foods rich in zinc are also high in phytic acid, a main inhibitor of zinc bioavailability [28]. Zinc bioavailability may be reduced by as much as 20% from a diet low in meat, and by as much as 50% in vegetarian diets high in phytates [29]. Zinc functions in the body as an intracellular molecule for immune cells [30], and zinc deficiencies may manifest as growth retardation, cognitive impairment, infection, and inflammation [31], as well as many chronic diseases as a result of the impaired immune response [32].

We found that majority of the participants consumed <70% the DRI for vitamins A and B-12, despite fortification. These nutrients are more bioavailable from ASFs and deficiencies are therefore most common among families who cannot afford, or do not consume, substantial amounts of eggs, dairy or meat products [33].

Our data show that more than 90% of the participants consume <70% DRI of calcium and vitamin D. The lack of calcium fortification in staple foods is a cause of concern in rural South Africa, as intake of dairy products is minimal, and typical diets contain large amounts of phytates, oxalates and tannins, which reduce calcium absorption [34]. In addition, dietary calcium absorption is reduced by as much as 20–30% when low meat diets are consumed (Venti & Johnston 2002). Inadequate intake of calcium/dairy promotes fat oxidation, favors a decrease in energy intake and facilitates of appetite control [35], [36]. Dietary calcium deficiency also has negative effects on vitamin D metabolism. Vitamin D deficiency has been correlated with obesity, diabetes mellitus, dyslipidemia, endothelial dysfunction and hypertension, all of which are factors increasing CVD risk [37].

Excess dietary sodium intake is estimated to be a leading health risk worldwide [38]. We found sodium intakes across all age and gender groups to exceed the DRI’s by 300 mg–700 mg. Results collected from a subsection of this study analyzing the nutritional composition of composite dishes in rural KwaZulu-Natal indicate that substantial amounts of salt are added during the cooking process for many composite dishes. High dietary salt is an important contributor to high blood pressure [39], and many conditions including stroke, coronary heart disease, and heart failure have been linked to the hormonal and cellular responses that result from excess dietary sodium intake [40].

Although South Africa has food composition data [41]–[44], we used the USDA database to maintain consistency with our other studies conducted around the globe [45]. Therefore, some bias may have occurred in estimating nutrient intakes using an American food database to calculate the nutritional composition of South African dishes. The USDA database, however has been shown to produce nutrient compositions that are highly correlated with data collected from direct chemical analysis [46].

The co-existence of over-nutrition and under-nutrition in South Africa [7] accentuates the need for careful and accurate assessment of dietary adequacy in specific population groups. It is essential to identify which nutrients are being consumed in excess, and which are being consumed below recommendations, and the health consequences of the current dietary pattern. Effective intervention programs designed to specifically target dietary insufficiencies can then be developed to improve the health outcomes of individuals in the target population.

### Conclusion

We have analyzed the dietary adequacy of the peoples of rural Empangeni, KwaZulu-Natal. Our results show that despite mandatory fortification of staple South African foods with many key micronutrients, the DRIs of vitamins A, B12, C, D, and E, calcium, zinc and pantothenic acid were not met by the majority of the population. In addition, calories, carbohydrates, and sodium were consumed in excess.
